# Frail Multiple Myeloma Patients Deserve More Than Just a Score

**DOI:** 10.3390/hematolrep15010015

**Published:** 2023-02-21

**Authors:** Hannah Louise Miller, Faye Amelia Sharpley

**Affiliations:** Christie @ Macclesfield, Macclesfield Hospital, Victoria Road, Macclesfield SK10 3BL, UK

**Keywords:** multiple myeloma, frailty, frailty score

## Abstract

Frailty is a hot topic in the field of multiple myeloma (MM). Clinicians have realised that frail myeloma patients can struggle with treatment, resulting in dose reductions and treatment discontinuation, which risk shorter progression-free and overall survival. Efforts have focused on the validity of existing frailty scores and on the development of new indices to identify frail patients more accurately. This review article explores the challenges of the existing frailty scores, including the International Myeloma Working Group (IMWG) frailty score, the revised Myeloma Co-morbidity Index (R-MCI), and the Myeloma Risk Profile (MRP). We conclude that the missing link is for frailty scoring to translate into a tool useful in real-world clinical practice. The future of frailty scores lies in their ability to be woven into clinical trials, to create a robust clinical evidence base for treatment selection and dose modification, and also to identify a cohort of patients who merit additional support from the wider MM multidisciplinary team.

## 1. Introduction

Multiple myeloma (MM) is a malignancy of the plasma cells [1] that predominately affects older people. MM is responsible for 15% of all haematological malignancies [2], and the incidence is increasing due to an ageing population.

The gold-standard treatment combines high-dose chemotherapy with autologous stem cell transplant (ASCT); however, for the majority, this high-intensity therapy is not feasible due to co-morbidities [3], frailty, and polypharmacy. Age is often used as a starting point to establish treatment decisions; however, this can lead to variations in therapy based on clinician perception [4]. Inconsistencies are highest for ‘fit elderly’ and ‘frail young’ patients, which highlights the important distinction between advanced age and frailty, but what exactly is frailty?

The British Geriatric Society defines frailty as ‘related to the ageing process, in which multiple body systems gradually lose their input reserves.’ Xue et al. expand on this definition describing frailty as “a clinically recognisable state of increased vulnerability resulting from an aging-associated decline in reserve and function across multiple physiologic systems, such that the ability to cope with every day or acute stressors is comprised [5].” Fried et al. opt for a functional assessment tool to identify those that are frail, defined by meeting three out of five of the following: low grip strength, low energy, slowed walking speed, low physical activity, and/or unintentional weight loss [6]. In a bid to improve on Freid’s definition, others have developed frailty indices and scores that combine co-morbidities and psychosocial risk factors in an attempt to more accurately predict adverse health outcomes.

Frailty scores are not just the remit of the geriatrician. MM-specific frailty scores exist (Table 1). The International Myeloma Working Group (IMWG) designed a frailty index based on age, the Charlson Comorbidity Index (CCI), Activities of Daily Living (ADL), and instrumental ADL (IADL) [7]. Patients defined as ‘frail’ with the IMWG frailty index have greater functional impairments and loss of muscle mass than ‘non-frail’ patients, indicating that the index reflects biological frailty. This tool can be used as a valuable starting point when considering degrees of frailty, although the lack of data on ADL and IADL in many studies has impeded the validation of the IMWG tool.

Cook et al. (2019) have created the UK Myeloma Research Alliance risk profile (MRP) to stratify MM patients’ ineligibility for stem-cell transplant [8]. This score incorporates ECOG performance status (PS), the Revised International Staging System (R-ISS), age, and C-reactive protein concentration to predict survival outcomes. The risk score, however, does not consider patient co-morbidities or functional testing [9]. It can therefore be used as a risk assessment tool but is unlikely to aid treatment decisions.

Two other MM-specific frailty tools have been developed: the Revised Myeloma Comorbidity Index (R-MCI) [10,11] and the Mayo Risk Score. However, due to the inclusion of pulmonary function tests and NT-proBNP, measures of lung and cardiac function, respectively, they are currently limited in their use, as this information is not routinely captured in MM patients.

## 2. The Current Use of Frailty Scores in Clinical Practice

The MM community widely accepts frailty as an important concept. Frail MM patients experience more adverse drug reactions [7], resulting in an inability to maintain treatment intensity and, in turn, poorer responses to therapy. That said, frailty scores have not yet been incorporated into routine clinical practice [12].

One of the issues appears to be around not just *which* tool to use, but *how* these frailty tools can be used to help guide clinical practice. Farcet et al. propose that geriatric assessment tools or frailty scores should be used to aid treatment decisions [13]. Zweegman et al. (2017) also advocate for the use of ‘geriatric scores’ to define fit, intermediate, and frail patients and tailor treatment accordingly. They suggest that the vast heterogeneity in fitness within the elderly population means there is a significant chance of over-treating of frail patients, leading to increased toxicity, discontinuation, and poorer treatment outcomes [14]. The authors go on to comment on the value of using frailty scores [7] based on a study by Cook et al. (2019) [8]; however, the study only considers their use in predicting patient outcomes and not frailty-adapted treatment. Many appreciate that the ultimate role of frailty scores should be to inform treatment choices, but this is yet to be determined [15].

Kaweme et al. conclude that although comprehensive geriatric assessment tools are available, there is limited evidence about their outcomes [16]. They suggest that although these tools are available, they are “time-consuming and challenging to use in everyday practice”.

## 3. The Future Potential of Frailty Scores

### 3.1. In the Trial Setting

Advancing therapies for patients with MM have largely improved disease outcomes, although the benefits are less clear-cut in frail patients [10]. Cook et al. explain this by suggesting that ‘patient factors’ are the probable cause for differences in treatment outcomes, as the incidence of ‘high-risk disease’ is the same in this cohort [10]. One such ‘patient factor’ would be frailty.

Clinical trials often discriminate against ‘unfit’ or ‘frail’ patients. Frail and unfit patients are all too often ‘excluded’ from entering clinical trials and subsequently suffer from over- or under-treatment [17].

**Figure 1:**
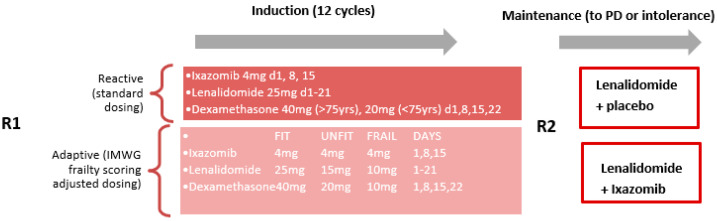
**Adapted from** The FiTNEss trial (Myeloma XIV) trial, which is a UK-MRA phase III, multi-centre, randomised controlled trial for transplant-ineligible, newly diagnosed MM patients. Patients are first randomised (R1) to standard (reactive) or frailty-adjusted (adaptive, based on IMWG score) with the triplet ixazomib, lenalidomide, and dexamethasone (IRd). A second randomisation (R2) compares progression-free survival with maintenance lenalidomide plus placebo (R) and lenalidomide plus ixazomib (IR). Adapted from Coulson BA et al. (2022) [18].

One of the most exciting applications of frailty scores is their incorporation into clinical trials, and one of the first trials to do this is the Frailty-adjusted Therapy in Transplant Non-Eligible patients with newly diagnosed Multiple Myeloma (FiTNEss (UK-MRA Myeloma XIV Trial)) [18]. This study is a randomised phase III trial that aims to investigate whether dose adjustments dependent on frailty will improve a patient’s ability to remain on therapy, reduce toxicity, and improve clinical outcomes (Figure 1). The hope is that trials such as this will open recruitment up to frail patients, allowing a better understanding of treatment toxicity and if discontinuation is the real reason behind their poorer outcomes, and additionally, finding solutions to reduce inequalities for older MM patients in the future.

### 3.2. In the Clinic Setting

**Figure 2:**
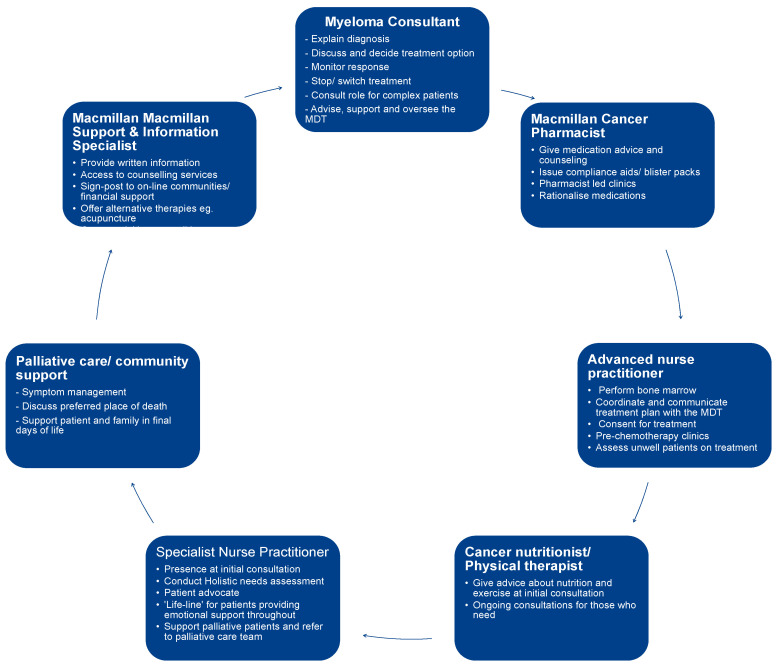
**Reprinted with permission from** A holistic myeloma clinic model of care, adapted from Sharpley FA et al. (2021) [19].

Another promising application of frailty scoring is the ability to identify a subset of MM patients who may benefit from additional support. At The Christie @ Macclesfield, we hope to use frailty scores to identify frail MM patients who would benefit from allied professional support. A pilot study, conducted by specialist oncology/haematology pharmacist Hannah Miller (yet unpublished), suggested that 48% of our MM patients on active treatment would be considered ‘frail’ using the IMWG score. The pilot study also revealed that 45% of patients reported medication-related issues, which may be in part due to polypharmacy (defined as taking four or more non-MM medications in addition to their anti-MM medication), as only 3% of patients took no additional medication other than those supplied by their MM clinician. Our aim at The Christie @ Macclesfield is to establish the UK’s first holistic MM clinic (Figure 2), where frail MM patients can access nutritional therapists, physiotherapists, specialist nurses, counsellors, and a Macmillan Cancer pharmacist. Our aim is to use a single frailty score to identify our MM patients most in need and introduce an intervention in the form of a holistic clinic experience to establish if we can reduce treatment toxicities, improve compliance, and reduce therapy discontinuation rates, ultimately leading to improved patient outcomes and satisfaction.

## 4. Discussion

Frailty is not equivalent to being old; not all old people are frail, and not all frail people are old, although advancing age is often associated with frailty and can equate to increased vulnerability. There is no gold-standard definition of frailty, leaving it open to interpretation and the development of increasingly complex scoring systems. The recent interest in frailty for patients with MM is due to the correlation with poor outcomes. Research to date has focused on revising and refining existing frailty scores to better predict prognosis and outcome, but has lost sight of why we are identifying these patients in the first place.

Palumbo’s IMWG frailty score, published in 2015 [7], was the first frailty score established specifically for MM. The IMWG score correctly focuses on what makes patients with MM frail: their biological vulnerability, due to their age and comorbidities, which is reflected through an inability to independently perform activities of daily living. The score weighs age heavily, which can mis-categorise the ‘fit elderly’ as frail and is arguably too time-consuming for routine use in clinical practice.

The FIRST trial is internationally recognised as one of the largest trials assessing the survival outcomes of transplant-ineligible, newly diagnosed MM. The phase 3 trial utilised a simplified frailty score based on age, CCI, and ECOG and was predictive of outcomes, with frail patients experiencing worse progression-free and overall survival. [20] However, both this score and other attempts to ‘improve’ Palumbo’s IMWG frailty score have lost sight of the fact that a frailty score should simply *identify* frail patients rather than predict survival outcomes. The fact that frail patients have adverse outcomes has confounded researchers, leading them to plug other adverse *disease*-related features (such as R-ISS stage and cytogenetics) into their algorithms. The aim was never just to identify all those MM patients with adverse outcomes but to identify those who are frail.

The lack of involvement of frailty scores in routine clinical practice highlights not only how confusing all the scores are for the jobbing MM clinician, but also how complex and time-consuming frailty scores are and how they are not appropriate for already busy myeloma clinics. Electronic frailty scores are helpful to this end; an example of this is described by DuMontier et al. [21].

A further barrier to the use of frailty scores may simply be that clinicians are unsure how the results can be practically applied to improve outcomes for their patients. Moller et al. (2021) should be commended for their attempt to translate a frailty score into a pragmatic treatment guide. The classification of MM patients as “go-go”, “intermediate-go”, and “slow-go” corresponds to a suggested starting dose of the most widely used anti-MM drugs [22]. The difficulty is that the evidence base for such starting doses is lacking.

A further pragmatic application of frailty scores is to identify patients who would benefit from more holistic support, as described. We appreciate that this requires the time and expertise of several allied health professionals, along with careful coordination and additional funding. For many centres, this model may be appealing, but not feasible in clinical practice in an already resource-poor and overstretched National Health Service (NHS).

The inclusion of frailty scores in large, national, and international trials is a promising way to improve awareness of the importance of frailty and illustrate how frailty scores can be applied in practice to help our patients with MM.

The most exciting adoption of frailty scoring to date has been by Coulson BA et al. (2022) in the FiTNEss UK-MRA Myeloma XIV trial, and we eagerly await the results of this study to see if a frailty score ‘adaptive’ approach to dosing is superior to ‘reactive’ dosing [18]. If this approach is successful, frailty scoring may become a standard way of dosing our non-transplant eligible patients in the future, which will hopefully open additional time and resources for frailty research in the future.

In the meantime, we stress the importance of remembering that frail patients need support, and this is not limited to frail patients with MM but all those deemed to be frail. Frail patients need identification to focus efforts on improving outcomes through the reduction of polypharmacy, the prompt management of side effects with supportive medications and supporting holistically to keep active, positive, and connected during treatment.

For us in the Christie @ Macclesfield, frailty scoring is not simply going to be used as an end in itself, but as means to an end for our patients. We will identify them as frail to provide them with more support and reduce their likelihood of treatment discontinuation, with the ultimate goal of improving their clinical outcomes. We hope that this paper has encouraged others to do the same.

## Figures and Tables

**Table 1:** A comparison of the main Myeloma-specific frailty scores; Table from Cook G, et al. *Leukemia*. (2020) [10].

Frailty Score	Biological Components	Functionality Tests	Comparison with IMWG	Populations Tested	Prospective Evaluation?
IMWG	Age, CCI	ADL, IADL	-	CT	No
R-MCI	eGFR, PFTs, Frailty, Age +/− CG	PS (Karnofsky)	Yes	CT, RW	Yes
UK MRA MRP	R-ISS, CRP, Age	PS (WHO)	No	CT, RW	No
Mayo Risk Score	NT-proBNP, Age	PS (WHO)	No	RW	No
Ancona Vulnerability Score	CCI	PS (WHO)	No	RW	No

ADL, activities of daily living; CCI, Charlson Comorbidity Index; CG, cytogenetics; CRP, C-reactive protein; CT, clinical trials; eGFR, estimated glomerular filtration rate; IADL, independent activities of daily living; IMWG, International Myeloma Working Group; R-ISS, revised International Staging System; NT-proBNP, N-terminal fragment of the type-B natriuretic peptide; R-MCI, revised Myeloma Comorbidity Index; RW, real world; PFTs, pulmonary function tests; PS, performance status; UKMRA MRP, UK Myeloma Research Alliance Myeloma Risk Profile; WHO, World Health Organization.

## Data Availability

Not applicable.
